# Trends in the Assessment of Social Frailty in Community-Dwelling Older Adults: A Scoping Review

**DOI:** 10.7759/cureus.66614

**Published:** 2024-08-10

**Authors:** Shinno Iijima, Akihiro Ito, Shomaru Ito, Takura Sasaki, Yuta Sugita

**Affiliations:** 1 Department of Physical Medicine and Rehabilitation, International University of Health and Welfare Hospital, Nasushiobara, JPN; 2 Department of Physical Therapy, School of Health Science, International University of Health and Welfare, Otawara, JPN; 3 Department of Physical Medicine and Rehabilitation, International University of Health and Welfare Narita Hospital, Narita, JPN; 4 Department of Physical Medicine and Rehabilitation, Aoikai Medical Corporation, Nasushiobara, JPN; 5 Department of Physical Therapy, Nishinasuno General Home Care Center, Nasushiobara, JPN

**Keywords:** mental health, scoping review, bunt classification, older adults, social frailty

## Abstract

Assessment of social frailty is crucial; however, definitions and assessment methods lack standardization. This review examined social frailty in community-dwelling older adults, highlighted trends in the definitions and assessment items used to date, and identified issues in assessing social frailty. The PubMed and CINAHL databases were searched for articles related to social frailty published up to 2022, and 95 articles were included in this review. The Bunt classification was used to assess the trends in items considered indicative of social frailty. Existing rating scales for social frailty were used in 82% of studies, and cut-off values were defined in 62% of studies. Factors such as the level of education; social interaction (weekly outings); and feelings of abandonment, emptiness, and lack of social integration (absence of a partner and non-participation in social organizations or activities) were evaluated less frequently. This study revealed that subjective feelings, including the fulfillment of social needs and participation in social activities, are less commonly considered in the assessment of social frailty.

## Introduction and background

Frailty is a geriatric syndrome defined as a state of increased vulnerability to stress owing to decreased reserve capacity in older adults. Frailty comprises several elements, including physical, psychological, and social components [1], with particular emphasis on the social component. Social frailty is often interpreted as the lack of resources to meet basic "social needs." Theories explaining social needs include the self-determination theory [2], loneliness theory [3], and social production function (SPF) theory [4]. Bunt et al. conducted a scoping review to structure and integrate social frailty based on the SPF theory. They extracted exhaustive components of social frailty, including subdivided elements from general resources, social resources, social behavior, and social needs fulfillment (Bunt classification) [5]. Briefly, the assessment of social frailty included the evaluation of older adults’ interactions with people around them, their living environment, their participation in social activities, and their environmental needs. Social frailty is problematic because of several reasons. For instance, a decrease in social interaction can trigger a deteriorating health status. Studies have reported a relationship between social frailty and the risk of developing diseases and depressive symptoms, and individuals with social frailty have a 2.31 times higher risk of developing depressive symptoms compared to those without social frailty [6,7]. Additionally, a longitudinal study on older adults reported significantly higher all-cause mortality in 10-12 years among individuals with a history of lack of conversation with neighbors and reduced community participation. This suggests that social frailty may negatively affect physical and mental health status [8-10]. Thus, social frailty is not only an expression of daily life and social isolation but is also considered a high-risk condition that causes mental and physical health problems in older adults. Makizako et al. evaluated older adults who did not exhibit physical frailty but were suspected of having social frailty, such as living alone, going out less frequently, and limited conversations with others for four years. They reported that the risk of developing new physical frailty was four times higher among older adults with elements of social frailty [11]. Therefore, accurate and early assessment of social frailty is crucial to provide necessary interventions before it leads to serious health problems. Despite the recognized importance of assessing social frailty, a standardized method for the early identification of these issues is lacking. Although various assessment scales exist, the choice of components to be assessed varies according to the researcher. Furthermore, social frailty may not be adequately assessed owing to the lack of clear cutoffs for determining social frailty in the literature. Although Bunt et al. conducted a scoping review to validate the construct of social frailty, no report has addressed the status of social frailty assessments in community-dwelling older adults [5]. Therefore, we conducted a scoping review of primary studies that assessed social frailty in community-dwelling older adults to identify the assessment scales and criteria used to determine social frailty. Additionally, we aimed to identify issues in the assessment of social frailty in community-dwelling older adults by highlighting trends in the elements extracted from each study based on Bunt et al.'s classification.

## Review

Material and methods

The PubMed and CINAHL electronic databases were searched for relevant studies. The search formula was "social frailty" AND (older OR elderly OR aged), and the search field was "All Fields" (search date: September 26, 2022). This report adheres to the Preferred Reporting Items for Systematic Reviews and Meta-Analyses extension for Scoping Reviews (PRISMA-Scr) guidelines (2018). The study was registered with the Open Science Framework in preparation for this paper (https://doi.org/10.17605/OSF.IO/3FTD5). The eligibility criteria were as follows: (1) studies assessing social frailty and (2) studies evaluating community-dwelling older adults (aged ≥60 years). The exclusion criteria were as follows: (1) the paper was not available, (2) the publication language was not English, (3) the article was not original, and (4) the study assessed hospitalized patients. Study eligibility was determined based on the title and abstract in the primary screening and information in the main text in the secondary screening. Screening was performed by two independent reviewers, and disagreements were resolved by a third reviewer. In articles selected after the screening, we assessed information on the use of existing assessment batteries, the classification of assessment components based on the Bunt classification, and the definition of social frailty. Studies were rated by two independent raters and discrepancies in ratings were resolved by a third rater. The results were summarized using descriptive statistics.

Bunt Classification

Social frailty has four components: general resources, social resources, social behavior, and satisfaction of social needs. These components are further subdivided as follows: General resources: living alone, level of education, lack of support, and financial difficulties; social resources: absence of a support person, neighbor, or close friend or presence of someone to rely on; social behavior: frequency of going out, social interaction, limitation of social activities, opportunities to talk to someone, visiting friends, social contact, frequency of contact with family and neighbors, frequency of contact with society, and lack of social integration; satisfaction of social needs: feelings of abandonment, emptiness, loneliness, or being useful to friends and family; lack of social relationships; and social support.

Results

**Figure 1:**
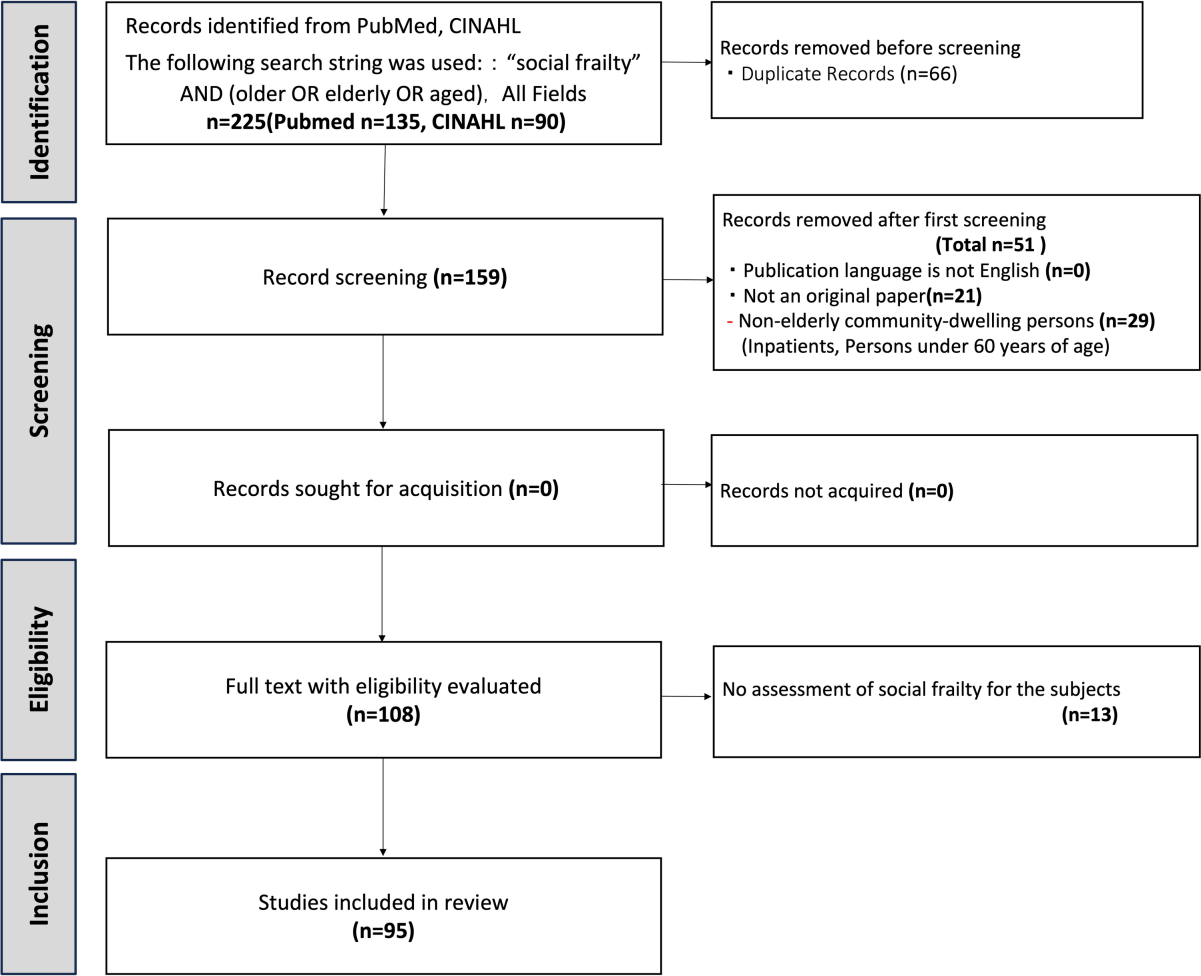
PRISMA flowchart of the study selection for the scoping review. PRISMA: Preferred Reporting Items for Systematic Reviews and Meta-Analyses

Of the 159 articles on social frailty published up to September 2022, 95 articles that evaluated community-dwelling older adults were included in this review. The inclusion of papers in the scoping review is shown in the flowchart (Figure 1). Studies excluded at the eligibility stage are presented in the appendix.

Study Design Trends

The 95 articles included 52 cross-sectional studies (55%), 21 longitudinal studies (22%), hybrid study (cross-sectional and longitudinal study, 3%), 11 prospective cohort studies (12%), two retrospective cohort studies (2%), two randomized controlled trials (2%), two prospective intervention studies (pre-/post comparison, non-randomized) (2%), one mixed-methods study (1%), and one case-control study (1%).

Social Frailty Assessment Index and Criteria

Fourteen percent (13/95) of the studies did not use an existing assessment battery to assess social frailty; instead, they used their own assessment methods. Existing assessment batteries were used in 82 studies (86%), and three studies (3%) used multiple batteries simultaneously. Sixty-one studies (64%) used an existing assessment battery and clearly defined a cutoff point for determining social frailty. Additionally, among the 13 studies that used an original method to assess social frailty, nine used a clear cutoff for determining social frailty (Table 1).

**Table 1:** Frequency of use of each rating scale, presence of criteria for social frailty, and content of the ratings. TFI, Tilburg Frailty Indicator; SFS-8, 8-item Social Frailty Scale; GFI, Groningen Frailty Indicator; LSNS-6, Lubben Social Network Scale-6; CFAI, Comprehensive Frailty Assessment Instrument; HALFT, Help, Participation, Loneliness, Financial, and Talk; KFS, Korean Frailty Scale; FGE, Functional Geriatric Evaluation

Rating Scale	Frequency of Use (Number of cases)	Existence of Judgment Criteria (Yes/No)	Factors Included in the Evaluation
TFI	34% (29)	15/29	Living alone, lack of social relationships, lack of social support
Makizako method	31% (27)	27/27	Living alone, frequency of going out, visiting friends, helping friends and family, talking every day
Yamada method	6% (5)	5/5	Living alone, financial difficulties, lack of social activities, frequency of contact with neighbors
SFS-8	3% (3)	3/3	Living alone, visiting friends, talking to family and friends, confidants, frequency of going out, eating with someone, financial constraints, daily conversation
GFI	3% (3)	1/3	Absence of neighbors, sense of abandonment, emptiness
LSNS-6	3% (3)	2/3	Number of family and friends (see/talk regularly, can talk privately, can ask for help)
CFAI	3% (3)	0/3	Social loneliness, social support
HALFT	2% (2)	2/2	Loneliness, support from others, limited social activities, financial difficulties, ability to talk to someone
KFS	2% (2)	1/2	Social network, social support
FGE	2% (2)	0/2	Social and economic resources

In studies that used existing assessment batteries, a total of 86 batteries were used; the Tilburg Frailty Indicator (TFI), Makizako's method, and Yamada's method were used in 29 (34%), 27 (31%), and 5 (6%) studies, respectively. The 8-item Social Frailty Scale, the Groningen Frailty Indicator, the Lubben Social Network Scale-6, and the Comprehensive Frailty Assessment Instrument were used in three studies (3%) each. The HALFT, Korean Frailty Scale, Functional Geriatric Evaluation, Fried score, PRISMA7, Frailty Phenotype, Clinical Frailty Scale, Biopsychosocial Frailty, University of California Los Angeles Loneliness Scale, California Los Angeles Loneliness Scale-3 items, and Modified Reported Edmonton Frail Scale were used in one study (1%) each.

Trends in Elements Classified as Social Frailty

**Figure 2:**
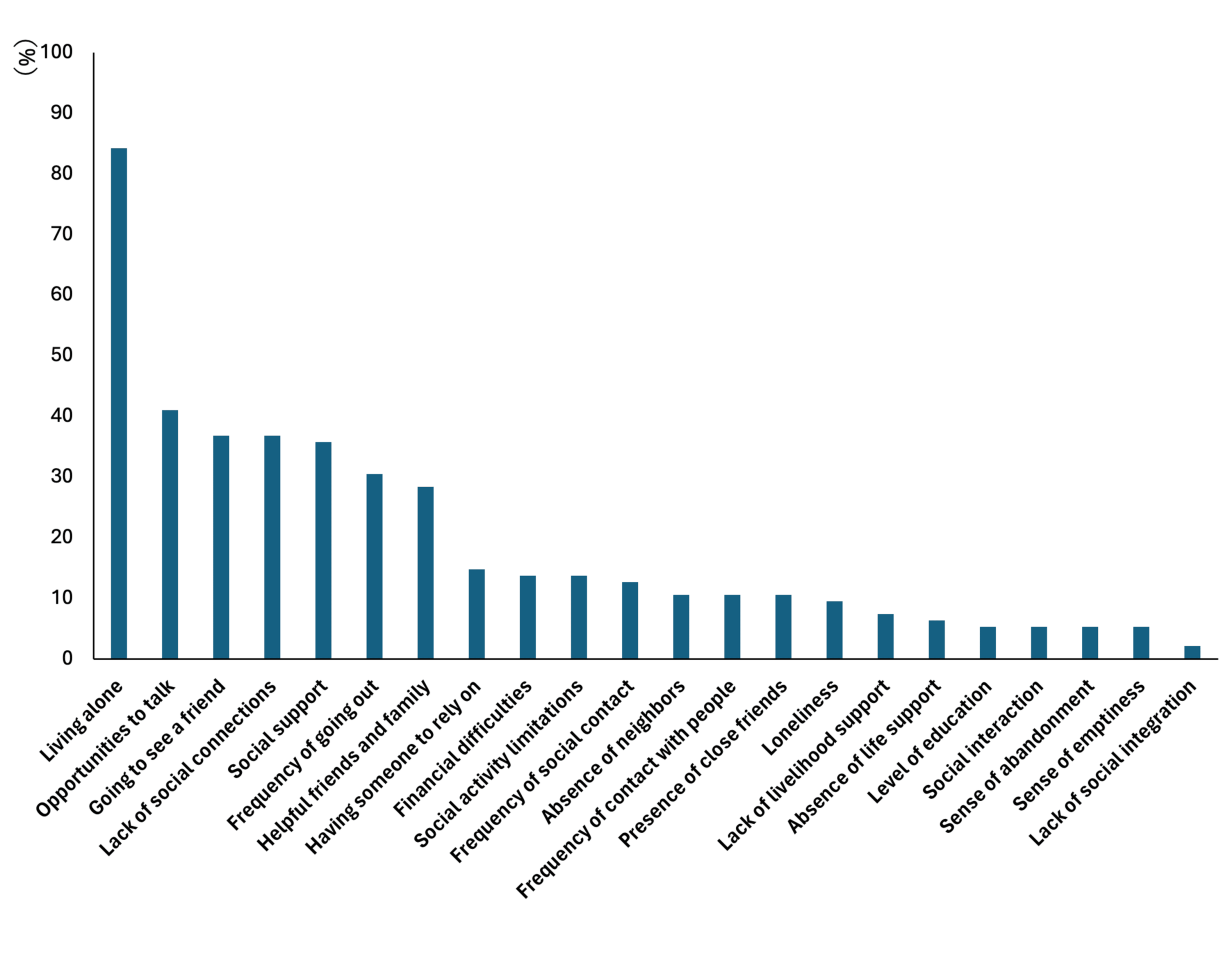
Proportions of elements classified as social frailty.

The most commonly evaluated factors according to the Bunt classification were living alone (85%, 80/95), having someone to talk to (41%, 39/95), visiting friends (37%, 35/95), lack of social relationships (37%, 35/95), lack of social support (37%, 34/95), going out frequently (31%, 29/95), being helpful to friends and family (28%, 27/95), and having someone to rely on (15%, 14/95). The least-evaluated factors were loneliness (9%, 9/95), lack of life support (7%, 7/95), lack of a life-support person (6%, 6/95), level of education (5%, 5/95), social interaction (5%, 5/95), feeling abandoned (5%, 5/95), feeling empty (5%, 5/95), and lack of social integration (2%, 2/95) (Figure 2).

Discussion

This scoping review examined current methods of assessment of social frailty in community-dwelling older adults. Additionally, trends in the current reporting of social frailty assessments and future issues were evaluated.

Rating Scales and Criteria for Social Frailty

In primary research on social frailty in community-dwelling older adults, existing assessment scales were used in > 80% of the studies. A total of 17 assessment scales were used, and no scale was established as a gold standard. The most commonly used scales were the TFI (34%) and the Makizako method (27%). The TFI is a frailty assessment tool developed by Gobbens et al. in the Netherlands and is divided into Part A, which indicates baseline characteristics, and Part B, which assesses frailty status. In Part B, 15 items, including physical, psychological, and social factors, are evaluated using a two-factor method, and a person is classified as frail if five or more items are applicable. The facts that the scale has been translated into several languages in several countries and that the validity of the overall assessment of frailty has been demonstrated (α = 0.79, κ = 0.79) are believed to contribute to the widespread use of the scale [12]. Social frailty is defined based on the sub-items of living alone, lack of social relationships, and lack of social support, but definite criteria for determining social frailty are lacking. In primary studies using TFI, social frailty was determined based on the presence of two of the three sub-items [13,14]. Makizako et al. [11] developed the Makizako method in Japan, which consists of five components: living alone, not going out frequently, not visiting friends, not being useful to friends and family, and not talking to someone daily. The presence of one of these factors is defined as presocial frailty, and the presence of two or more factors is considered social frailty [15,16]. Other scales, such as the Yamada index [17] and SFI [18], are assessment methods based in part on the items listed in Bunt's scoping review, using items related to social needs satisfaction, social resources, general resources, and social behaviors and activities. Seventy-two percent of the studies using the Yamada scale were reported from Japan, suggesting a regional bias. Among the included studies, 60% used some type of cutoff to determine social frailty. Forty percent of the studies did not specify which items were used to determine social frailty or simply used scale scores for group comparisons but tended not to examine the clinical implications of the scores. In other words, authors tended to make relative assessments of social frailty, which may lead to arbitrary judgments and increase the risk of publication bias. The challenge is to clarify the criteria that should be used to assess social frailty. This is expected to lead to a multidisciplinary understanding of social frailty and the development of a gradation of social frailty.

Trends and Challenges in Assessing Social Frailty Factors

The most frequently asked question in the assessment of social frailty was "Does the patient live alone?" The importance of "living alone" in the assessment of frailty has long been debated. A series of studies on social support by Makizako et al. [7,11,15,19] reported living alone as an independent factor for social frailty. However, Sakurai et al. found that the presence of a roommate did not directly affect health status; nonetheless, poor social networking was the background for deterioration in health [20]. Although the mechanism by which living alone induces frailty has not been fully elucidated [21], it should be evaluated in terms of whether support in times of need, such as from distant family members or neighborhood social services, can be easily obtained in the long term. The "Kashiwa Study (Japan)" of community-dwelling older adults reported that attention should be paid to the association between oral frailty (frailty related to dental and oral functions) and social frailty [22]. In that study, participants who ate alone had less communication with family members, even if they did not live alone, and many of them had unbalanced diets and poor nutritional status. In other words, older adults who are isolated from their families may be at the threshold of physical and social frailty, even though they do not live alone; assessment of such cases is challenging. In general, the elements corresponding to the "satisfaction of social needs" tended to be evaluated less often. These elements evaluate how the individual feels in society and their expectations from the outside world. However, Bunt et al. reported that assessment of subjective aspects of social frailty was difficult [5]. In a previous study, subjective factors, such as loneliness and alienation, which are difficult to assess objectively, tended to be excluded as they reduced the validity of the scale when creating rating scales. In addition, the items that extract the subjects’ feelings might include aspects of psychological frailty, and because the boundary between psychological and social frailty is difficult to grasp, assessment of these items is difficult. However, regarding assessment for the early detection of the need for the use of social resources and the introduction of services, the subjective factor of the type of assistance required should be evaluated as a check for social frailty. This study identified key components of social frailty, such as the frequency of social interactions and the presence of support networks, which can be directly influenced by the types of assistance individuals perceive they need.

A limitation of this study is that it did not evaluate studies other than those included in the databases used in this study. The amount of information obtained can be increased by broadening the scope of the search. Based on the results of this scoping review, we suggest the following: First, research reports should clearly describe the criteria for determining social frailty using existing or original rating scales. A clear presentation of the scoring criteria will lead to a multidisciplinary interpretation of the information. In addition, we believe that adding subjective factors, such as individual values and thoughts, to the actual assessment in community and clinical settings will facilitate the detection of social frailty risks. This approach will allow for a more comprehensive understanding of the social aspects influencing frailty. Therefore, in the future, supplementing the information by combining subjective factors that are difficult to express using existing rating scales with qualitative assessments, such as interviews, should be considered. It is necessary to conduct new studies to establish a gold standard for the assessment of social frailty.

## Conclusions

This review showed that 60% of the studies used cutoffs to assess social frailty, and biases in the establishment of the cutoffs were evident. Particularly, subjective feelings and participation in social activities to fulfil social needs were infrequently included in the assessment of social frailty. Clarification of missing information will help identify signs of social frailty at earlier stages. Future studies should focus on defining the criteria for the evaluation of frailty and establishing a gold standard for assessment.

## Appendices

**Table 2:** The PRISMA-ScR checklist. JBI = Joanna Briggs Institute; PRISMA-ScR = Preferred Reporting Items for Systematic Reviews and Meta-Analyses extension for Scoping Reviews. * Where sources of evidence (see second footnote) are compiled from, such as bibliographic databases, social media platforms, and websites. † A more inclusive/heterogeneous term used to account for the different types of evidence or data sources (e.g., quantitative and/or qualitative research, expert opinion, and policy documents) that may be eligible in a scoping review as opposed to only studies. This is not to be confused with information sources (see first footnote). ‡ The frameworks by Arksey et al. (6) and Levac et al. (7) and the JBI guidance (4, 5) refer to the process of data extraction in a scoping review as data charting. § The process of systematically examining research evidence to assess its validity, results, and relevance before using it to inform a decision. This term is used for items 12 and 19 instead of "risk of bias" (which is more applicable to systematic reviews of interventions) to include and acknowledge the various sources of evidence that may be used in a scoping review (e.g., quantitative and/or qualitative research, expert opinion, and policy document). From: Tricco AC, Lillie E, Zarin W, O'Brien KK, Colquhoun H, Levac D, et al. PRISMA Extension for Scoping Reviews (PRISMAScR): checklist and explanation. Ann Intern Med. 2018;169:467-73. doi: 10.7326/M18-0850.

SECTION	ITEM	PRISMA-ScR CHECKLIST ITEM	REPORTED ON PAGE #
TITLE			
Title	1	Identify the report as a scoping review.	1
ABSTRACT			
Structured summary	2	Provide a structured summary that includes (as applicable): background, objectives, eligibility criteria, sources of evidence, charting methods, results, and conclusions that relate to the review questions and objectives.	1
INTRODUCTION			
Rationale	3	Describe the rationale for the review in the context of what is already known. Explain why the review questions/objectives lend themselves to a scoping review approach.	1
Objectives	4	Provide an explicit statement of the questions and objectives being addressed with reference to their key elements (e.g., population or participants, concepts, and context) or other relevant key elements used to conceptualize the review questions and/or objectives.	1
METHODS			
Protocol and registration	5	Indicate whether a review protocol exists; state if and where it can be accessed (e.g., a Web address); and if available, provide registration information, including the registration number.	2
Eligibility criteria	6	Specify characteristics of the sources of evidence used as eligibility criteria (e.g., years considered, language, and publication status), and provide a rationale.	2
Information sources*	7	Describe all information sources in the search (e.g., databases with dates of coverage and contact with authors to identify additional sources), as well as the date the most recent search was executed.	2
Search	8	Present the full electronic search strategy for at least 1 database, including any limits used, such that it could be repeated.	2
Selection of sources of evidence†	9	State the process for selecting sources of evidence (i.e., screening and eligibility) included in the scoping review.	2
Data charting process‡	10	Describe the methods of charting data from the included sources of evidence (e.g., calibrated forms or forms that have been tested by the team before their use, and whether data charting was done independently or in duplicate) and any processes for obtaining and confirming data from investigators.	2
Data items	11	List and define all variables for which data were sought and any assumptions and simplifications made.	2
Critical appraisal of individual sources of evidence§	12	If done, provide a rationale for conducting a critical appraisal of included sources of evidence; describe the methods used and how this information was used in any data synthesis (if appropriate).	-
Synthesis of results	13	Describe the methods of handling and summarizing the data that were charted.	2
RESULTS			
Selection of sources of evidence	14	Give numbers of sources of evidence screened, assessed for eligibility, and included in the review, with reasons for exclusions at each stage, ideally using a flow diagram.	Figure1
Characteristics of sources of evidence	15	For each source of evidence, present characteristics for which data were charted and provide the citations.	appendix
Critical appraisal within sources of evidence	16	If done, present data on critical appraisal of included sources of evidence (see item 12).	-
Results of individual sources of evidence	17	For each included source of evidence, present the relevant data that were charted that relate to the review questions and objectives.	Table1
Synthesis of results	18	Summarize and/or present the charting results as they relate to the review questions and objectives.	3-4
DISCUSSION			
Summary of evidence	19	Summarize the main results (including an overview of concepts, themes, and types of evidence available), link to the review questions and objectives, and consider the relevance to key groups.	4
Limitations	20	Discuss the limitations of the scoping review process.	5
Conclusions	21	Provide a general interpretation of the results with respect to the review questions and objectives, as well as potential implications and/or next steps.	5
FUNDING			
Funding	22	Describe sources of funding for the included sources of evidence, as well as sources of funding for the scoping review. Describe the role of the funders of the scoping review.	6

**Table 3:** List of studies included in this research.

No	Title	Authors	Citation
1	Social Frailty in Community-Dwelling Older Adults as a Risk Factor for Disability	Makizako et al.	J Am Med Dir Assoc. 2015, 16(11):1003.e7-11.
2	Social Frailty Is Associated with Physical Functioning, Cognition, and Depression, and Predicts Mortality	Ma et al.	J Nutr Health Aging.2018;22(8):989-995.
3	Reciprocal Relationship Between Locomotive Syndrome and Social Frailty in Older Adults	Ono et al.	Geriatr Gerontol Int.2021, 21(11):981-984.
4	Social Frailty and Depression Among Older Adults in Ghana: Insights from the WHO SAGE Surveys	Amegbor et al.	Res Aging. 2021, 43(2):85-95.
5	Association Between Social Frailty and Sleep Quality among Community-Dwelling Older Adults: A Cross-Sectional Study	Noguchi et al.	Phys Ther Res. 2021, 24(2):153-162.
6	Association Between Oral, Social, and Physical Frailty in Community-Dwelling Older Adults	Hironaka et al.	Arch Gerontol Geriatr. 2020, 89:104105.
7	Interventions Aimed at Loneliness and Fall Prevention Reduce Frailty in Elderly Urban Population	Ožić et al.	Medicine (Baltimore). 2020, 99(8):e19145.
8	Association of Sleep Condition and Social Frailty in Community-Dwelling Older People	Nakakubo et al.	Geriatr Gerontol Int. 2019, 19(9):885-889.
9	Social Frailty Predicts Incident Disability and Mortality Among Community-Dwelling Japanese Older Adults	Yamada et al.	J Am Med Dir Assoc. 2018, 19(12):1099-1103.
10	Association of Social Frailty With Both Cognitive and Physical Deficits Among Older People	Tsutsumimoto et al.	J Am Med Dir Assoc. 2017, 18(7):603-607.
11	Social Frailty Dimensions and Frailty Models Over Time	Bessa et al.	Arch Gerontol Geriatr. 2021, 97:104515.
12	Association Between Social Frailty as well as Early Physical Dysfunction and Exercise Intolerance Among Older Patients Receiving Hemodialysis	Usui et al.	Geriatr Gerontol Int. 2021, 21(8):664-669.
13	Association of Social Frailty with Physical Health, Cognitive Function, Psychological Health, and Life Satisfaction in Community-Dwelling Older Koreans	Ko et al.	Int J Environ Res Public Health. 2021, 18(2):818.
14	Factors Associated with Physical, Psychological and Social Frailty Among Community-Dwelling Older Persons in Europe: A Cross-Sectional Study of Urban Health Centres Europe (UHCE)	Ye et al.	BMC Geriatr. 2021, 21(1):422.
15	Sex-Specific Association between Social Frailty and Diet Quality, Diet Quantity, and Nutrition in Community-Dwelling Elderly	Huang et al.	Nutrients. 2020, 12(9):2845.
16	Evaluation of Bio-Psycho-Social Frailty in Older Persons on the Territory: The Method and the Experience of the "Medesano Health House"	Gelmini et al.	Acta Biomed. 2020, 91(2):389-395.
17	Frailty and Quality of Life Among Community-Dwelling Older Adults	Papathanasiou et al.	Cureus. 2021, 13(2):e13049.
18	Impact of Social Frailty on Relocation of Older Adults	Dupuis-Blanchard et al.	J Frailty Aging. 2021;10(3):254-258.
19	Association Between Physical, Psychological and Social Frailty and Health-Related Quality of Life Among Older People	Zhang et al.	Eur J Public Health. 2019, 29(5):936-942.
20	Associations of Social Frailty with Loss of Muscle Mass and Muscle Weakness Among Community-Dwelling Older Adults	Makizako et al.	Geriatr Gerontol Int. 2019, 19(1):76-80.
21	Multidimensional and Physical Frailty in Elderly People: Participation in Senior Organizations Does Not Prevent Social Frailty and Most Prevalent Psychological Deficits	Sacha et al.	Front Public Health. 2020, 8:276.
22	Social Frailty and Health-Related Quality of Life in Community-Dwelling Older Adults	Ko et al.	Int J Environ Res Public Health. 2022, 19(9):5659.
23	Multimorbidity, Trauma Exposure, and Frailty of Older Adults in the Community	Papathanasiou et al.	Front Genet. 2021, 12:634742.
24	Impact of Social Frailty on the Association Between Driving Status and Disability in Older Adults	Doi et al.	Arch Gerontol Geriatr. 2022, 99:104597.
25	Screening Value of Social Frailty and Its Association with Physical Frailty and Disability in Community-Dwelling Older Koreans: Aging Study of PyeongChang Rural Area	Park et al.	Int J Environ Res Public Health. 2019, 16(16):2809.
26	The Role of Social Frailty in Explaining the Association Between Hearing Problems and Mild Cognitive Impairment in Older Adults	Bae et al.	Arch Gerontol Geriatr. 2018, 78:45-50.
27	Moderate Hearing Loss Is Related with Social Frailty in a Community-Dwelling Older Adults: The Korean Frailty and Aging Cohort Study (KFACS)	Yoo et al.	Arch Gerontol Geriatr. 2019, 83:126-130.
28	Associations Between Obesity and Multidimensional Frailty in Older Chinese People with Hypertension	Song et al.	Clin Interv Aging. 2020, 15:811-820.
29	Social Frailty and Depressive Symptoms During the COVID-19 Pandemic Among Older Adults in Japan: Role of Home Exercise Habits	Hayashi et al.	Arch Gerontol Geriatr. 2022, 98:104555.
30	Clinical Characteristics and Social Frailty of Super-Elderly Patients With Heart Failure - The Kitakawachi Clinical Background and Outcome of Heart Failure Registry	Takabayashi et al.	Circ J. 2016, 81(1):69-76.
31	The Relationship Between the House Boundedness and Frailty of Community-Dwelling Elderly Persons	Katsura et al.	J Rural Med. 2018, 13(2):141-150.
32	Influence of Combined Cognitive Impairment and Social Frailty on Physical Frailty in Community-Dwelling Older Adults	Ko et al.	Geriatr Nurs. 2022, 46:125-131.
33	Association Between Social Capital and Frailty and the Mediating Effect of Health-Promoting Lifestyles in Chinese Older Adults: Across-Sectional Study	Hu et al.	BMC Geriatr. 2022, 22(1):175.
34	Multidimensional Frailty and Pain in Community Dwelling Elderly	Coelho et al.	Pain Med. 2017, 18(4):693-701.
35	Longitudinal Association Between Physical Activity and Frailty Among Community-Dwelling Older Adults	Zhang et al.	J Am Geriatr Soc. 2020, 68(7):1484-1493.
36	The Association of Social Frailty with Intrinsic Capacity in Community-Dwelling Older Adults: A Prospective Cohort Study	Huang et al.	BMC Geriatr. 2021, 21(1):515.
37	Social Frailty and Executive Function: Association with Geriatric Syndromes, Life Space and Quality of Life in Healthy Community-Dwelling Older Adults	Ong et al.	J Frailty Aging. 2022;11(2):206-213.
38	Prediction of Mortality by the Tilburg Frailty Indicator (TFI)	Gobbens et al.	J Am Med Dir Assoc. 2021, 22(3):607.e1-607.e6.
39	Frailty Differences in Older Adults' Use of Informal and Formal Care	Lambotte et al.	Arch Gerontol Geriatr. 2018, 79:69-77.
40	Osteosarcopenia, the Co-existence of Osteoporosis and Sarcopenia, Is Associated with Social Frailty in Older Adults	Inoue et al.	Aging Clin Exp Res. 2022, 34(3):535-543.
41	Association Between Satisfaction with Meaningful Activities and Social Frailty in Community-Dwelling Japanese Older Adults	Miyata et al.	Arch Gerontol Geriatr. 2022, 100:104665.
42	Development and Validation of a Multidimensional Frailty Scale for Clinical Geriatric Assessment	Shin et al.	J Nutr Health Aging. 2021;25(7):938-943.
43	Physical Frailty Predicts the Development of Social Frailty: A Prospective Cohort Study	Nagai et al.	BMC Geriatr. 2020, 20(1):403.
44	Social Frailty and Longitudinal Risk of Depressive Symptoms in a Chinese Population: The Rugao Longevity and Aging Study	Chen et al.	Psychogeriatrics. 2021, 21(4):483-490.
45	The Predictive Value of Social Frailty on Adverse Outcomes in Older Adults Living in the Community	Lee et al.	J Am Med Dir Assoc. 2020, 21(10):1464-1469.e2.
46	Frailty Related to the Exposure to Particulate Matter and Ozone: The Korean Frailty and Aging Cohort Study	Shin et al.	Int J Environ Res Public Health. 2021, 10;18(22):11796.
47	Prevalence, Overlap, and Interrelationships of Physical, Cognitive, Psychological, and Social Frailty Among Community-Dwelling Older People in Japan	Sugie et al.	Arch Gerontol Geriatr. 2022, 100:104659.
48	Preoperative Frailty and Outcome in Patients Undergoing Radical Cystectomy	van der Vlies et al.	BJU Int. 2020,126(3):388-395.
49	The Prediction of Quality of Life by Physical, Psychological and Social Components of Frailty in Community-Dwelling Older People	Gobbens et al.	Qual Life Res. 2014, 23(8):2289-300.
50	Effect of a Robotic Pet on Social and Physical Frailty in Community-Dwelling Older Adults: A Randomized Controlled Trial	Pollak et al.	Res Gerontol Nurs. 2022, 15(5):229-237.
51	Social Frailty and Functional Disability: Findings From the Singapore Longitudinal Ageing Studies	Teo et al.	Studies. J Am Med Dir Assoc. 2017, 18(7):637.e13-637.e19.
52	Associations Between Multidimensional Frailty and Quality of Life Among Dutch Older People	Gobbens et al.	Arch Gerontol Geriatr. 2017, 73:69-76.
53	Cross-Sectional and Longitudinal Associations of Environmental Factors with Frailty and Disability in Older People	Gobbens	Arch Gerontol Geriatr. 2019, 85:103901.
54	Chronic Pain Is Independently Associated with Social Frailty in Community-Dwelling Older Adults	Hirase et al.	Geriatr Gerontol Int. 2019, 19(11):1153-1156.
55	Determinants of Frailty	Gobbens et al.	J Am Med Dir Assoc. 2010, 11(5):356-64.
56	Social Frailty Is Independently Associated with Mood, Nutrition, Physical Performance, and Physical Activity: Insights from a Theory-Guided Approach	Pek et al.	Int J Environ Res Public Health. 2020, 17(12):4239.
57	Relationship of Frailty Markers and Socioeconomic Status to Incidence of Depressive Symptoms in a Community Cohort	Lian et al.	J Am Med Dir Assoc. 2021, 22(3):570-576.e1.
58	Are Physically Frail Older Persons at Risk of Adverse Outcomes If They Also Suffer From Cognitive, Social, and Psychological Frailty?	Ament et al.	Eur J Ageing. 2014, 11(3):213-219.
59	Being Your Best: Protocol for a Feasibility Study of a Codesigned Approach to Reduce Symptoms of Frailty in People Aged 65 Years or More After Transition From Hospital	Lowthian et al.	BMJ Open. 2021, 11(3):e043223.
60	Meaningful Activities and Psychosomatic Functions in Japanese Older Adults after Driving Cessation	Nakamura et al.	Int J Environ Res Public Health. 2021, 18(24):13270.
61	Links Between Physical Frailty and Regional Gray Matter Volumes in Older Adults: A Voxel-Based Morphometry Study	Nishita et al.	J Am Med Dir Assoc. 2019, 20(12):1587-1592.e7.
62	Impact of childhood trauma on multidimensional frailty in older patients with a unipolar depressive-, anxiety- or somatic symptom disorder	Schmahl et al.	Arch Gerontol Geriatr. 2021, 96:104452.
63	Social Frailty Has a Stronger Impact on the Onset of Depressive Symptoms than Physical Frailty or Cognitive Impairment: A 4-Year Follow-up Longitudinal Cohort Study	Tsutsumimoto et al.	J Am Med Dir Assoc. 2018, 19(6):504-510.
64	Reinforcement Effects of Social Network Intervention Suring Nutritional Supplementation in Frail Older Adults	Kim et al.	Gerontology. 2021, 67(5):620-632.
65	Development of a Clinical Support System for Identifying Social Frailty	Kuo et al.	Int J Med Inform. 2019, 132:103979.
66	Prevalence of Frailty Phenotypes and Risk of Mortality in a Community-Dwelling Elderly Cohort	Garre-Olmo et al.	Age Ageing. 2013, 42(1):46-51.
67	Effects of Frailty and Chronic Diseases on Quality of Life in Dutch Community-Dwelling Older Adults: A Cross-Sectional Study	Renne et al.	Clin Interv Aging. 2018, 13:325-334.
68	Simultaneous Employment of the FRAIL Scale and the Tilburg Frailty Indicator May Identify Elderly People Who Require Different Interventional Strategies	Sacha et al.	Clin Interv Aging. 2020, 15:683-690.
69	Social Frailty Leads to the Development of Physical Frailty Among Physically Non-Frail Adults: A Four-Year Follow-Up Longitudinal Cohort Study	Makizako et al.	Int J Environ Res Public Health. 2018, 15(3):490.
70	Co-existence of Social Isolation and Homebound Status Increase the Risk of All-Cause Mortality	Sakurai et al.	Int Psychogeriatr. 2019, 31(5):703-711.
71	Impact of Social Frailty on Alzheimer's Disease Onset: A 53-Month Longitudinal Cohort Study	Tsutsumimoto et al.	J Alzheimers Dis. 2019, 70(2):587-595.
72	Characteristics of Meaningful Activities in Community-Dwelling Japanese Older Adults with Pre-frailty and Frailty	Maruta et al.	Arch Gerontol Geriatr. 2022, 99:104616.
73	The Effect of Community Nurse on Mortality and Hospitalization in a Group of Over-75 Older Adults: A Nested Case-Control Study	Terracciano et al.	Ann Ig. 2021, 33(5):487-498.
74	Predictors of Emergency Room Access and Not Urgent Emergency Room Access by the Frail Older Adults	Gentili et al.	Front Public Health. 2021, 9:721634.
75	Translation, Adaptation, and Reliability of a Social Frailty Scale for the Brazilian Context: A Methodological Study	Damasceno et al.	Sao Paulo Med J. 2022, S1516-31802022005021218.
76	Frailty as a Predictor of Short-Term Adverse Outcomes	Coelho et al.	PeerJ. 2015, 3:e1121. PMID: 26246968; PMCID: PMC4525687.
77	Intradialytic Exercise in the Treatment of Social Frailty: A Single-Center Prospective Study-Preliminary Results During the Unexpected COVID-19 Pandemic	Abe et al.	Ren Replace Ther. 2020, 6(1):36.
78	The Influence of Multiple Frailty Profiles on Institutionalization and All-Cause Mortality in Community-Living Older Adults	Lee et al.	J Cachexia Sarcopenia Muscle. 2022, 13(5):2322-2330.
79	Associations Between Lifestyle Factors and Multidimensional Frailty: A Cross-Sectional Study Among Community-Dwelling Older People	van Assen et al.	BMC Geriatr. 2022, 22(1):7.
80	Frailty Affects Self-Care Behavior in Congestive Heart Failure	Li et al.	Clin Nurs Res. 2022, 31(4):615-623.
81	Assessing Timewise Changes Over 15 Months in Life-Space Mobility Among Community-Dwelling Elderly Persons	Hayashi et al.	BMC Geriatr. 2020, 20(1):502.
82	Social Frailty is Independently Associated with Geriatric Depression Among Older Adults Living in Northern Japan: A Cross-Sectional Study of ORANGE Registry	Kume et al.	Geriatr Gerontol Int. 2022, 22(2):145-151.
83	Social Frailty as a Risk Factor for New-Onset Depressive Symptoms at One-Year Post-Surgery in Older Patients with Gastrointestinal Cancer	Okumura et al.	J Geriatr Oncol. 2020, 11(5):904-907.
84	Determinants of Frailty: The Added Value of Assessing Medication	Coelho et al.	Front Aging Neurosci. 2015, 7:56.
85	A Cross-Sectional Study on the Different Domains of Frailty for Independent Living Older Adults	Verver et al.	BMC Geriatr. 2019, 19(1):61.
86	Biopsychosocial Frailty and the Risk of Incident Dementia: The Italian Longitudinal Study on Aging	Solfrizzi et al.	Alzheimers Dement. 2019, 15(8):1019-1028.
87	Work Status Before Admission Relates to Prognosis in Older Patients with Heart Failure Partly Through Social Frailty	Yamashita et al.	J Cardiol. 2022, 79(3):439-445.
88	Social Frailty Increases the Risk of All-Cause Mortality: A Longitudinal Analysis of the English Longitudinal Study of Ageing	Ragusa et al.	Exp Gerontol. 2022, 167:111901.
89	The Simplified Nutritional Appetite Questionnaire (SNAQ) as a Screening Tool for Risk of Malnutrition: Optimal Cutoff, Factor Structure, and Validation in Healthy Community-Dwelling Older Adults	Lau et al.	Nutrients. 2020, 12(9):2885.
90	Validation of the Clinical Frailty Scale Version 2.0 in Turkish Older Patients	Aşık et al.	Geriatr Gerontol Int. 2022, 22(9):730-735.
91	Explaining Quality of Life of Older People in the Netherlands Using a Multidimensional Assessment of Frailty	Gobbens et al.	Qual Life Res. 2013, 22(8):2051-61.
92	Explaining Discrepancies in Self-reported Quality of Life in Frail Older People: A Mixed-Methods Study	van der Vorst et al.	BMC Geriatr. 2017, 17(1):251.
93	Impact of COVID-19 Pandemic Exacerbation of Depressive Symptoms for Social Frailty from the ORANGE Registry	Kodama et al.	Int J Environ Res Public Health. 2022, 19(2):986.
94	A Comparison of Different Modeling Techniques in Predicting Mortality With the Tilburg Frailty Indicator: Longitudinal Study	van der Ploeg et al.	JMIR Med Inform. 2022, 10(3):e31480.
95	Towards a More Effective Strategy to Detect Community-Dwelling Frail Older Adults: Validation of Risk Factors	Van der Elst et al.	INT J HEALTH GOV.2021,26(3)

**Table 4:** Excluded studies after eligibility verification.

No	Title	Authors	Citation	Reason for Exclusion
1	Social and Societal Implications of Frailty, Including Impact on Canadian Healthcare Systems.	Andrew et al.	J Frailty Aging. 2018;7(4):217-223.	Submitted as a review or opinion piece, not as an original study.
2	Consensus on Components of Frailty Using the Delphi Method: Korean Frailty and Aging Cohort Study.	Kim et al.	J Nutr Health Aging. 2021;25(2):242-247.	Study aimed at constructing a research model; survey conducted on experts.	
3	Key Stakeholders' Views on the Quality of Care and Services Available to Frail Seniors in Canada.	Giguere et al.	BMC Geriatr. 2018 Nov 26;18(1):290.	Qualitative study involving patients, healthcare and care workers; did not directly assess social frailty.
4	Assessing the Impact of a Community-Based Pro-active Monitoring Program Addressing the Need for Care of Community-Dwelling Citizens Aged More than 80: Protocol for a Prospective Pragmatic Trial and Results of the Baseline Assessment.	Liotta et al.	Transl Med UniSa. 2020 Dec 31;23:22-27.	Not focused on investigating social frailty.
5	Adaptation of a Social Vulnerability Index for Measuring Social Frailty Among East African Women.	Prabhu et al.	BMC Public Health. 2022 Jan 24;22(1):167.	Included a design that involved patients with diseases.
6	Social Frailty as Social Aspects of Frailty: Research, Practical Activities, and Prospects.	Fujiwara et al.	Geriatr Gerontol Int. 2022 Dec;22(12):991-996.	Submitted as a review or opinion piece, not as an original study.
7	Exploring Perceived Challenges of Self‐management in Low‐Income Older People with Hypertension: A Qualitative Study.	Zhang et al.	Int J Nurs Pract. 2022 Jun;28(3):e13059.	Included a design that involved patients with diseases.
8	Long-Term Life Care at Home: A Bottom up, Community-Driven Model for Long-Term Care Reform in Canada.	Giosa et al.	First North American Conference on Integrated Care.	Was an opinion piece and did not assess social frailty in subjects.
9	Adaptation of a social vulnerability index for measuring social frailty among East African women.	Prabhu et al.	BMC Public Health. 2022 Jan 24;22(1):167.	Study aimed at validating the evaluation method.
10	Beyond Physical Impairment: The Role of Social Frailty in Heart Failure.	Keshvani et al.	J Am Heart Assoc. 2021 Sep 7;10(17):e022187.	Was an opinion piece and did not assess social frailty in subjects.
11	COVID-19 Pandemic Control Measures: Impact on Social Frailty and Health Outcomes in Non-Frail Community-Dwelling Older Adults.	Pek et al.	J Nutr Health Aging. 2021;25(6):816-818.	Was an opinion piece and did not assess social frailty in subjects.	
12	Key stakeholders' views on the quality of care and services available to frail seniors in Canada.	Giguere et al.	BMC Geriatr. 2018 Nov 26;18(1):290.	Included a design that involved patients with diseases.
13	Falling through the cracks: a case study of how a timely integrated approach can reverse frailty.	Freer	Br J Community Nurs. 2020 Aug 2;25(8):382-387.	Was a case report on a single subject and did not validate the assessment of social frailty.
